# Rituximab Efficacy during a Refractory Polyarteritis Nodosa Flare

**DOI:** 10.1155/2009/738293

**Published:** 2010-03-14

**Authors:** Emmanuel Ribeiro, Thomas Cressend, Pierre Duffau, Marieke Grenouillet-Delacre, Marie Rouanet-Larivière, Anne Vital, Maïté Longy-Boursier, Patrick Mercié

**Affiliations:** ^1^Service de Médecine Interne, Hôpital Saint-André, Centre Hospitalier Universitaire de Bordeaux, Bordeaux, France; ^2^Service de Médecine Interne et Maladies Infectieuses, Hôpital Haut-Lévêque, Centre Hospitalier Universitaire de Bordeaux, Pessac, France; ^3^Service d'Exploration Fonctionnelle du Système Nerveux, Hôpital Pellegrin, Centre Hospitalier Universitaire de Bordeaux, Bordeaux, France; ^4^Laboratoire d'Anatomo-Pathologie, Hôpital Pellegrin, Centre Hospitalier Universitaire de Bordeaux, Bordeaux, France; ^5^Université Victor-Segalen Bordeaux 2, 146, rue Léo-Saignant, 33076 Bordeaux Cedex, France

## Abstract

Polyarteritis nodosa (PAN) is a systemic vasculitis whose severe forms are treated with glucocorticoids and cyclophosphamide. Refractory patients are exposed to many complications, notably accelerated atherosclerosis. We report a case report of 71-year-old man followed for polyarteritis nodosa refractory to glucocorticoids and cyclosphosphamide. Systemic vasculitis relapses are followed to accelerated atherosclerosis: severe ischemic lesions led to amputation of lower limbs. Remission of refractory PAN is obtained with rituximab. Disappearance of biological inflammatory is allowed to regression of ischemic lesions in upper limbs. In this situation, we recommend a systematic vascular work-up for patients suffered from refractory vasculitis. On the other hand, therapeutic trials are needed to determine the real efficacy and place of rituximab in the treatment of polyarteritis nodosa.

## 1. Presentation

Polyarteritis nodosa (PAN) is a systemic vasculitis of the medium-sized vessels whose severe form, treated with glucocorticoids and cyclophosphamide, can give rise to late complications, notably accelerated atherosclerosis, which remains poorly described in this disease. Remission of our patient's severe PAN flare, refractory to conventional treatment and complicated by accelerated atherosclerosis, was obtained with rituximab.

## 2. Assessment

**Figure 1:**
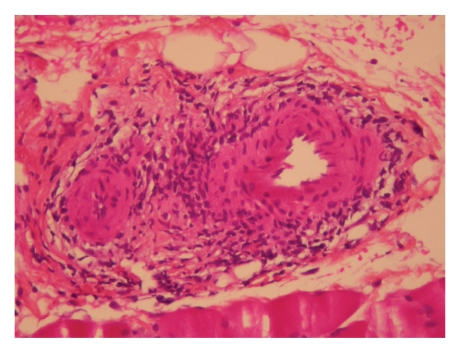
Vasculitis of a muscle arteriole with fibrinoid necrosis (magnification: ×250).

A 71-year-old man, with no remarkable medical history, had been followed for 2 years for systemic vasculitis with successive flares manifested by polyarthritis, skin lesions, and bilateral orchitis associated with a biological inflammatory syndrome without immunological anomalies (no antinuclear or antineutrophil cytoplasm antibodies (ANCAs) detected). When a relapse occurred under glucocorticoids, intravenous cyclophosphamide was started. After two cyclophosphamide pulses, the patient's condition deteriorated with subacute polyarthritis, myalgias, livedo and acrocyanosis of the four limbs. Signs of sensory-motor polyneuropathy were observed on electromyograms of the legs. Histological examination of a neuromuscular biopsy found vasculitis with fibrinoid necrosis compatible with, confirming the diagnosis of PAN (Figure 1). 

 Despite intravenous methylprednisolone, an acute coronary syndrome occurred with thrombosis of the right coronary artery, followed by acute ischemia of the left leg. Echo-Doppler ultrasonography detected occlusions of the left anterior and posterior and the right posterior tibial arteries. Echocardiography found no intracardiac thrombus. The persistent biological inflammatory syndrome (C-reactive protein (CRP), 79 mg/L) raised the possible risk of ischemic complications of the vasculitis in the legs. The inefficacy of conventional therapy led to the prescription of a weekly rituximab infusion (375 mg/m^2^/week for 4 weeks). The immediate worsening of the ischemic lesions, insensitive to all medical treatments, led to amputation of the lower left leg. Histopathological examination of the surgical specimen found atherosclerotic lesions in medium-sized vessels but no signs of vasculitis or cholesterol emboli, leading us to suspect accelerated atherosclerosis during a vasculitis flare in this patient with no known history of cardiovascular or atheromatous disease. 

 Rituximab was replaced by mycophenolate mofetil (1 g twice daily) combined with glucocorticoids. The outcome was favorable, with complete healing of the stump and rapid regression of the biological inflammatory syndrome. He secondarily developed, independently of any signs of vasculitis, a suprainfection of the big right toe that deteriorated rapidly. Despite rituximab being effective, amputation of the second lower leg was required. 

 The patient was again hospitalized 6 months later for necrotic skin lesions on the fingers on the left hand accompanied by the reappearance of the biological inflammatory syndrome (CRP, 58 mg/L). Echo-Doppler ultrasonography and arteriography of the arms found atheromatous plaques in the brachiocephalic trunk and occlusion of the left cubital and right radial arteries. 

 The coexistence of medium-sized vessel lesions and reappearance of the biological inflammatory syndrome led us to suspect a PAN relapse. Rituximab (375 mg/m^2^/week) was administered for 4 weeks. Rapid and complete healing of the digital ischemic lesions was observed with regression of the biological inflammatory syndrome (erythrocyte sedimentation rate, 28 mm/1st h; CRP, 17 mg/L). 

 Rituximab was continued as maintenance therapy, with a monthly infusion for 6 months followed by prolongation of the interinfusion interval to 2 months. At present, after 2 years, the patient is asymptomatic, and no new ischemic lesions have appeared. He continues to receive rituximab every 3 months and his general condition is good.

## 3. Discussion

Rituximab is a chimeric monoclonal antibody that selectively depletes CD20^+^ B lymphocytes. It is currently used to treat diverse autoimmune diseases and refractory vasculitides: rheumatoid arthritis (RA), systemic lupus erythematosus (SLE), Sjögren's syndrome, ANCA-associated vasculitides, mixed cryoglobulinemias, autoimmune cytopenias and neuropathies, myasthenia gravis, and bullous dermatoses [1]. Rituximab has also been successfully used to treat refractory Wegener's granulomatosis (WG) and microscopic polyangiitis (MPA) resistant to conventional regimens. Keogh et al. reported obtaining remissions in their 10 WG and one MPA patients with rituximab [2]. 

 Two case reports have suggested that rituximab is also effective against corticosteroid resistant polyarteritis nodosa [3, 4]. This rituximab efficacy against systemic inflammatory diseases led us to use this biologic twice to treat our patient's refractory PAN. It is difficult to explain the precise role of rituximab in the first remission because of the quasisimultaneous administration of cyclophosphamide and methylprednisolone pulses. In contrast, the second remission obtained with rituximab strongly suggests its efficacy at controlling the vasculitis.

Improved survival of patients whose severe and progressing systemic vasculitides continue to progress under maximal immunosuppressive therapy now enables us to see late complications that have previously been poorly described, notably the probable occurrence of accelerated atherosclerosis. 

 Accelerated atherosclerosis has mainly been studied in RA and SLE patients. Indeed, RA patients have suffered excess mortality of cardiovascular origin, with standardized all-cause-mortality ratios of 1.41 for women and 1.51 for men, and respective cardiovascular values of 2.02 and 1.34 [5]. Indeed, 30% of SLE deaths are of cardiovascular origin [6], with a relative risk of myocardial infarction multiplied by 5 [7]. 

 Unlike the autoimmune diseases addressed above, accelerated atherosclerosis in systemic vasculitides is rare, because it has been less studied. After adjustment for cardiovascular risk factors and CRP, it was recently demonstrated that small-sized-vessel vasculitides were associated with a higher frequency of subclinical atheromas [8]. Seyahi et al. [9] showed that their 30 patients with Takayasu arteritis had a significantly higher rate of subclinical atheromas than SLE patients and the healthy controls (*P* < .001). In another study on 29 WG patients, the intima-media thickness, a surrogate marker of atherosclerosis, was significantly (*P* < .05) wider compared to a group of controls with the same cardiovascular risk factors [10]. Concerning the pathophysiology of these lesions, the diffuse endothelial dysfunction present during the course of vasculitides [11] could facilitate the rapid development of atherosclerosis. 

 Concerning our patient, distinguishing between accelerated atherosclerosis and the specific vasculitis involvement was initially difficult. The histopathological examination of the amputated limbs confirmed the existence of atherosclerotic lesions of medium-sized vessels with no sign of associated PAN. It should be noted that the distribution of atherosclerotic lesions affecting medium-sized vessels was superimposable on the sites of predilection of inflammatory PAN lesions.

## 4. Conclusion

Among the new biotherapies potentially active against vasculitides resistant to conventional treatment, rituximab is given to treat severe, uncontrolled ANCA-associated vasculitides but it could also have a role to play in refractory PAN. Therapeutic trials are needed to determine the real efficacy and place of rituximab in the treatment of PAN. In these patients whose life expectancy can be prolonged by these new therapeutic options, it is possible to observe, during vasculitis flares, the appearance of potentially life-threatening accelerated atherosclerosis. The chronic or acute inflammation during vasculitis is probably a key element in the progression of atheromatous disease [12]. In these situations, it seems logical to recommend a systematic vascular work-up for patients with progressive and conventional treatment-resistant vasculitides.
